# Two patients with allergy to celery — Possible role of carbohydrate determinants and difference between seeds and tuber allergenicity

**DOI:** 10.1016/j.waojou.2022.100708

**Published:** 2022-11-07

**Authors:** Pawel Dubiela, Piotr Humeniuk, Merima Bublin, Carine Metz-Favre, Sébastien Viel, Francoise Bienvenu, Christine Hafner, Gabrielle Pauli, Karin Hoffmann-Sommergruber

**Affiliations:** a Department of Regenerative Medicine and Immune Regulation, Medical University of Bialystok, Bialystok, Poland; b Department of Pathophysiology, Medical University of Vienna, Vienna, Austria; c Department of Rheumatology and Inflammation, University of Gothenburg, Gothenburg, Sweden; d Allergy Division, Department of Chest Diseases, University Hospital, Strasbourg, France; e Immunology Laboratory, Hospices Civils de Lyon, Lyon, France; f Department of Dermatology, University Hospital St. Poelten, Karl Landsteiner University of Health Sciences, St. Poelten, Austria; g Medical University of Strasbourg, Strasbourg, France

**Keywords:** CCDs, Celery, Celery allergy, Component resolved diagnosis, Food allergy

## Abstract

Vegetables provide important nutrients but can also induce allergic symptoms. Celery tuber allergy frequently occurs in Central European countries and can cause allergic reactions including fatal anaphylactic shocks. There is little information about allergen content in seeds. Therefore, we analyzed 2 patients with allergic reaction after remoulade sauce consumption who entered the clinic for a diagnostic work-up. The routine diagnostic included serum derived specific IgE testing by ImmunoCAP, ImmunoCAP ISAC, and skin prick tests (SPTs). Furthermore, protein extracts were prepared from both celery tuber and celery seeds and IgE binding capacity of these extracts was assessed by immunoblots, ELISA, and rat basophil leukemia (RBL) assay. We also determined role of cross-reactive carbohydrate determinants (CCDs) by IgE inhibition ELISA. Results revealed distinct protein patterns from celery tuber and seed extracts, suggesting differences in content and quantity of allergenic proteins. IgE antibodies from both sera bound to high molecular weight (HMW) proteins on immunoblots and caused high basophil response, which was also observed upon addition of glycosylated proteins as horseradish peroxidase and Api g 5, respectively. Our results indicate that it is worth considering CCDs from plant foods as a possible allergenic factor and their contribution to the mugwort-celery syndrome.

Sensitization to celery tuber is one of the clinically important causes of food allergies, especially in Central European countries such as Germany, France, and Switzerland.^1^, ^2^, ^3^ A Swiss Study with 402 food allergic patients, showed IgE reactivity to celery in 42% of the patients.^4^ Birch pollen allergic patients can develop symptoms after celery consumption due to IgE cross reactivity between Api g 1 and the major birch pollen allergen, Bet v 1. Both allergens share 40% amino acid sequence identity and 60% similarity, respectively.^5^ It is a well-described example of the pollen-food allergy syndrome, and explains why more than 70% of birch pollen allergic patients have adverse reactions after consumption of some fruits, vegetables, and nuts.^6^^,^^7^ In contrast to the birch pollen-food syndrome, information on the mugwort-celery-syndrome is lacking and relevant cross-reactive allergens are not yet identified. It is highly possible that a causative celery protein homologous to the major mugwort allergen Art v 1 is still missing, and/or these cross-reactions are due to more than one allergen.

It has been shown, that native Api g 5 is able to induce basophil activation. In contrast to the glycosylated form, deglycosylated Api g 5 lacks binding activity of IgE from celery allergic patients’ sera.^8^ Sensitization to carbohydrate structures was detected in 38% of patients with celeriac allergy.^8^ Carbohydrate residues present on proteins are known as a strong IgE binding components, but their capability of eliciting allergic symptoms has been questioned^9^ as perfectly summarized by Hils et al.^10^ This protein-linked carbohydrate structures are responsible for cross-reactivity of sera from allergic patients among a wide range of allergenic proteins from plants and insects. The structural analysis showed that cross-reactive carbohydrate determinants (CCDs)-specific IgE antibodies are directed to ubiquitous plant glycans-(1,3)-fucose and/or (1,2)-xylose containing epitopes.^11^ In general, CCDs are regarded as IgE binding components of low clinical relevance.

Although a number of celery allergens were identified and are commonly used in *in vitro* diagnostic tests, it seems that the panel of celery allergens remains incomplete. In this study we report on 2 allergic patients, who experienced clinical symptoms after consumption of celery tuber. Testing sera from celery allergic patients enabled us to analyze the relevance of IgE antibodies directed against carbohydrate determinants that can have impact on the allergic reactions. Also the potential impact of CCDs to the mugwort-celery IgE cross reactions was investigated. Furthermore, we analyzed the allergen repertoire present in celery seed and tuber protein extracts by immunoblots.

Skin prick tests (SPTs) for food allergens were performed using frozen crude extracts (home-made) and SPT with pollens were performed with commercial extract (ALK-Abello, Hørsholm, Denmark). A positive skin reaction was defined as a mean wheal diameter ≥3 mm. Specific IgE to Ambrosia, Artemisia, nArt v 1, rApi g 1, rPhl p 1 was measured using the ImmunoCAP (Thermo Fisher Scientific, Goteborg, Sweden) with a cut off of 0.35 kUA/L. Additionally, sera were subjected to ImmunoCAP ISAC analysis (Thermo Fisher Scientific). IgE levels >0.3 ISU-E were considered as positive. The study was approved by the ethics committees of Lower Austria (GS4-EK-4/242-2013) and from the French Ministère de la Recherche (DC-2010-1095). Written informed consents were received from all individuals.

Celery seed and tuber protein extracts were obtained as previously described.^5^ Briefly, celery seeds and fragmented tuber were frozen, ground and proteins were extracted with 4 vol of PBS buffer, pH 7, at 4 °C stirring overnight. Extracts were separated by 15% SDS-PAGE gel (100 μg of proteins per line), and subsequently transferred to nitrocellulose membrane (0.2 μm). Membranes were blocked with 3% BSA and incubated overnight with diluted 1:20 sera from celery allergic patients. Detection of bound human IgE was performed with subsequent incubation of 125I-labelled anti-human IgE (BSM Diagnostica, Vienna, Austria) diluted 1:15 overnight at room temperature followed by autoradiography.

IgE inhibition ELISA was performed as follows: celery allergic patients’ sera (1:20 dilutions) were blocked overnight at 4 °C with 1, 10 and 50 μg/ml of Bet v 1, Api g 1, Api g 5, Art v 1, HRP, celery tuber and seed extract (self-inhibition control), respectively. MaxiSorp plates were coated with 100 μg/ml of celery seed extract. Plates were blocked with 3% BSA and incubated overnight with blocked sera from celery allergic patients. After washing, plates were incubated with AP-conjugated anti-human IgE antibodies, develop with AP substrate and measure optical density (OD) 405. Tests were performed in duplicates and results calculated as percentage of inhibitions.

RBL assay was performed as previously described.^12^ Briefly, RS-ATL8 cells were plated onto a 96-well plate and incubated for 3 h at 37 °C. Then, diluted sera were added to the supernatant (final dilution 1:100). Following overnight incubation, cells were stimulated for 3 h at 37 °C, with proteins, Api g 1, Api g 5, Bet v 1, HRP respectively, and celery tuber and seed extracts. Luciferase expression levels correspond to the fold of the light units increase compared with the background signal. All tests were done in triplicates.

Two allergic patients with reaction after remoulade sauce consumption entered the clinic for a diagnostic work-up. Remoulade sauce, a typical French sauce, is based on mayonnaise, vinegar, mustard, pickles, and celery. Clinical data of the patients (Table 1) indicated that celery could be the trigger of the reactions.

**Table 1:** Clinical data from 2 patients with celery allergy

	Age	Gender	Clinical symptoms	Positive SPT	Immuno CAP	ImmunoCAP ISAC 112 Multiplex Phadia (ISU)																		
						rAln g 1	rApi g 1	rV Api m 1	nV Api m 2	rAra h 8	nArt v 1	rBet v 1	rCor a 1	nCyn d 1	rGly m 4	nJug r 2	rMal d 1	rPhl p 1	nPhl p 4	rPhl p 5	rPhl p 6	nPla a 2	rPol d 5	rVes v 5
**Patient 1**	64	M	spring pollinosis urticaria - raw celeryoral allergy syndrome – cooked celery and parsley	raw/cooked celery, parsley,raw carrot, dandelion, fennel, cumin, artemisia, ambrosia, grass pollen, birch pollen	ambrosia: 19,6kU/lartemisia: 19,7kU/lApi g 1: <0.1kU/lArt v 1: 15.2kU/2Art v 3: <0.1kU/lAmb a 1: 0.14kU/lBet v 1: 0.2kU/lBet v 2: <0.1kU/l			1.05	1.79		1.15			5.13		1.19			5.14	1.25		0.38		
**Patient 2**	55	F	urticaria - raw celeryanaphylaxis - remoulade sauce	raw celery, artemisia, grass pollen, birch pollen	nArt v 1: 1.52 kU/lnApi g 1: 1.40 kU/lrPhl p 1: 80 kU/l	9.34	0.76			4.10	0.78	30.22	2.75	17.66	0.78		4.98	25.57	10.53	13.13	3.60		0.47	0.90

Patient number 1, a 64 year-old male, was suffering from pollinosis and allergy to parsley and both raw (urticaria) and cooked celery (oral allergy syndrome). SPT was positive to raw/cooked celery, parsley, raw carrot, dandelion, fennel, cumin, artemisia, ambrosia, grass, and birch pollen. ImmunoCAP tests were positive for Ambrosia (19.6 kU/l), Artemisia (19.7 kU/l) and Art v 1 (15.2 kU/I), respectively.

Patient number 2, a 55 year-old female also experienced urticaria upon consumption of raw celery but she did not report any symptoms to cooked celery. She was also suffering from spring pollinosis and oral syndrome with raw carrot. SPT was positive for raw celery, Artemisia, grass and birch pollen. ImmunoCAP was positive for nArt v 1 (1.52 kU/l), rApi g 1 (1.40 kU/l), and rPhl p 1 (80 kU/l), respectively.

Furthermore, patients’ sera were tested by ImmunoCAP ISAC 112 Multiplex Phadia. Serum from patient number 1 predominantly had specific IgE directed against glycosylated allergens. Serum from patient number 2 contained IgE antibodies against Bet v 1 homologues (Bet v 1, Aln g 1, Mal d 1, Cor a 1, and Api g 1), grass pollen allergens such as Cyn d 1, and major mugwort allergen, Art v 1.

Based on the clinical data we decided to investigate the IgE binding patterns of both sera for celery tuber and seed extracts. Celery tuber and seed extracts revealed different patterns of proteins in Coomassie stained 15% SDS-PAGE analysis (Fig. 1). Tuber extract contained more proteins in the high molecular mass range (HMW) as compared to the seed extract with more intense protein bands in the low molecular mass range.

**Fig. 1:**
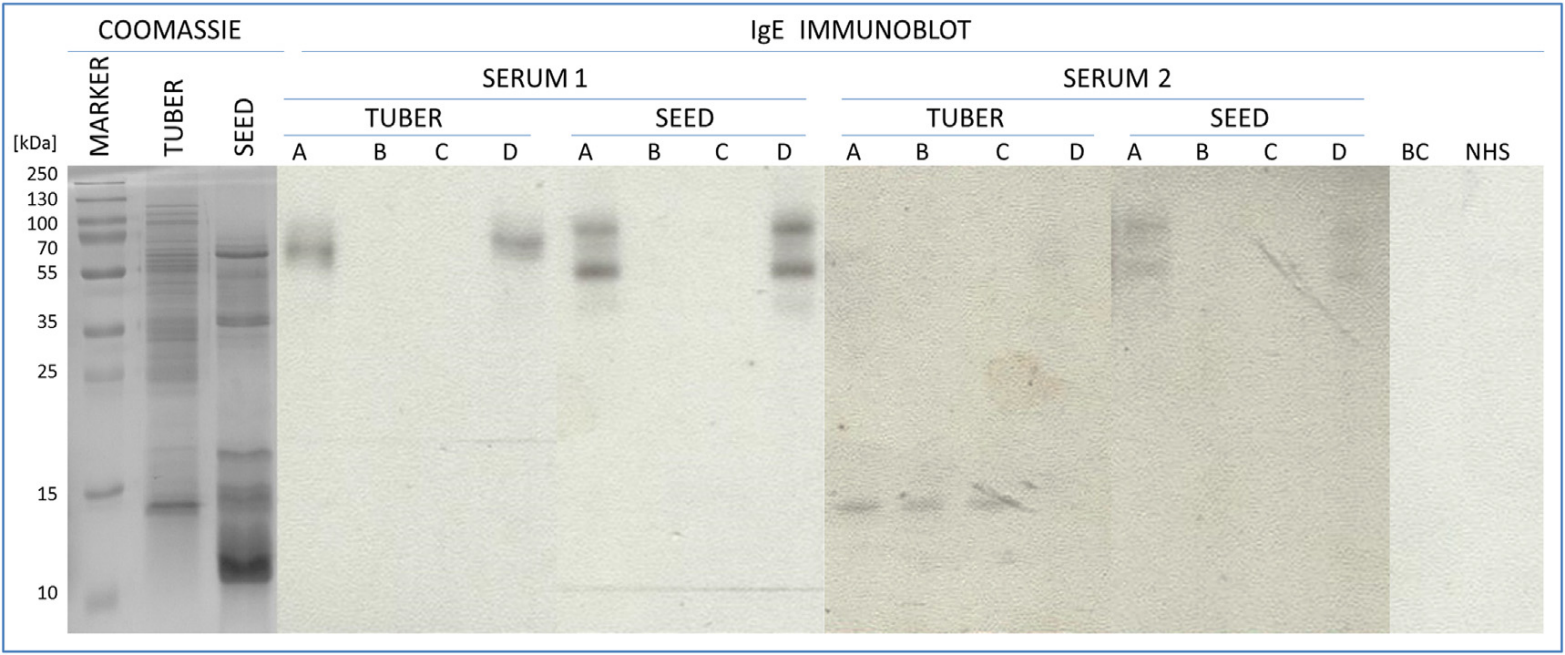
**Coomassie staining and IgE Immunoblot of celery tuber and seed extracts**. For IgE immunoblots sera from two allergic patients were tested: A, non inhibited serum, B, C, D, serum preincubated with HRP, nApi g 5 and rApi g 1 respectively; BC, buffer control; NHS, non allergic human serum control.

IgE immunoblots performed with celery seed extract provided a similar pattern when testing both sera. Specific IgE antibodies bound to 2 HMW proteins of 55 kDa and 90 kDa, respectively. Inhibition with glycosylated proteins, horseradish peroxidase (HRP) and nApi g 5, respectively, completely blocked IgE binding. In contrast, preincubation of sera with rApi g 1 did not affect the IgE recognition pattern.

Celery tuber extract contained only one HMW protein (65 kDa) recognized by IgE from serum of patient number 1 and incubation with HRP and Api g 5, blocked this binding capacity, whereas inhibition with Api g 1 did not affect this interaction. Serum from patient number 2 contained IgE specific to a protein of molecular mass of approximately 15 kDa, which was inhibited by preincubation of rApi g 1. This result was in line with the data from ImmunoCAP ISAC analysis. Blocking with HRP and nApi g 5 did not affect the IgE against rApi g 1, but depleted a weak signal from HMW proteins.

IgE inhibition ELISA experiments with the celery seed extract confirmed the previous results from immunoblot analyses. Specific IgE from serum number 1 recognized seed proteins and was completely inhibited by HRP and nApi g 5, but not by rApi g 1. Preincubation with nArt v 1, displayed more than 25% of inhibition. Blocking with a celery tuber extract showed almost complete inhibition (85% at a concentration of 50 μg/ml) (Fig. 2A). Serum from patient number 2 displayed IgE binding to celery seed proteins and preincubation with HRP completely blocked this binding. Natural Api g 5 and nArt v 1 in the highest concentrations (50 μg/ml) blocked the majority of specific antibody recognition (80%). Also pre-incubation of the serum with the celery tuber extract (50 μg/ml) resulted in 72% inhibition (Fig. 2B).

**Fig. 2:**
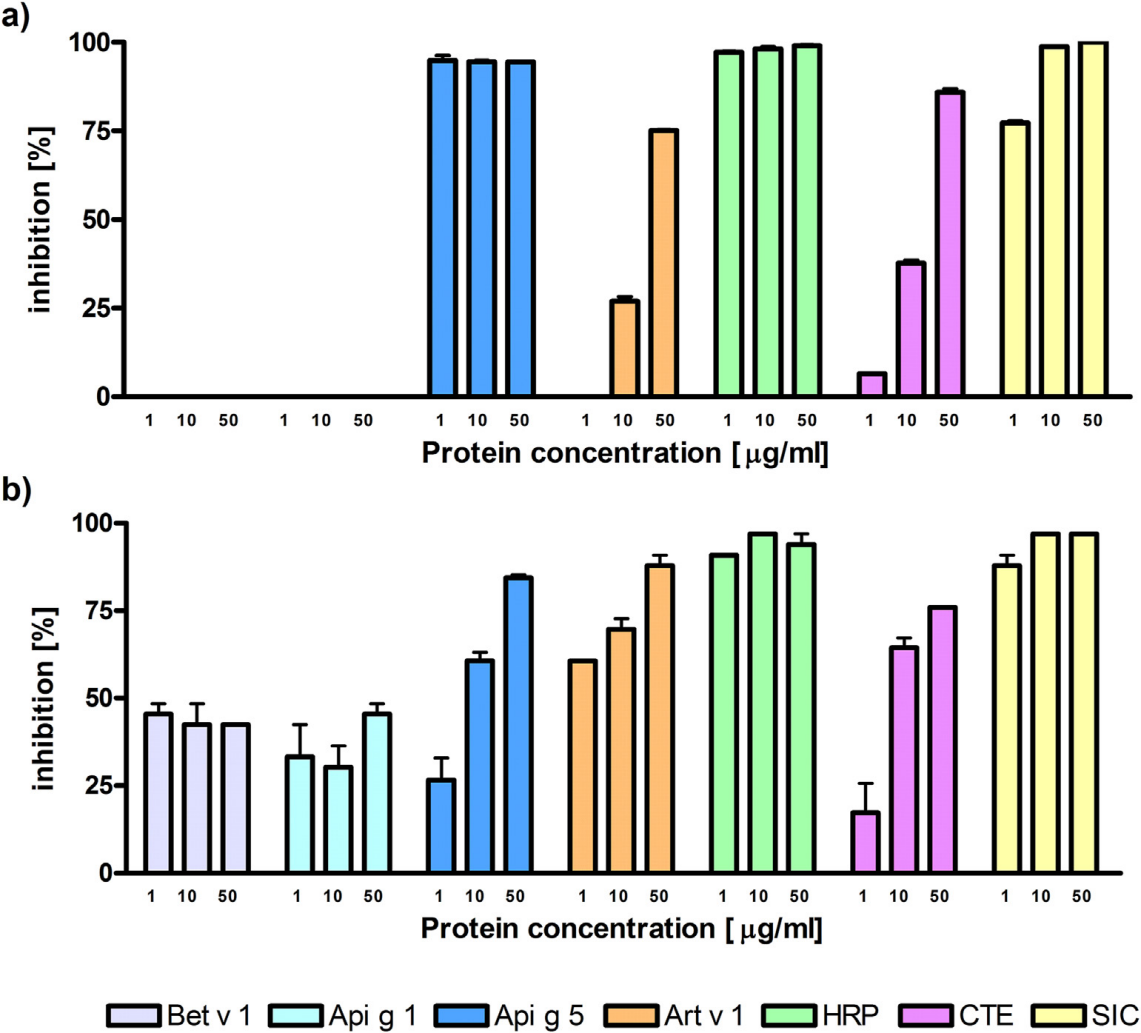
**IgE inhibition ELISA**. ELISA plates were coated with celery seed extract. Sera from patient 1 and 2 (panel a and b) were blocked with the panel of proteins in 3 different concentrations. Bars are presenting percentage of inhibitions. HRP, horseradish peroxidase; CTE, celery tuber extract; SIC, self inhibition control.

ELISA plates were coated with celery seed extract. Sera from patient 1 and 2 (panel a and b) were blocked with the panel of proteins in 3 different concentrations. Bars are presenting percentage of inhibitions. HRP - horseradish peroxidase; CTE – celery tuber extract; SIC – self inhibition control.

Due to the comparatively high OD values to glycosylated proteins observed in the IgE ELISA experiments we further investigated whether CCDs could activate basophils *in vitro*. Rat basophil leukemia (RBL) luciferase assay was performed with RS-ATL8 cells as previously described.^12^ Serum nr 1 induced high basophil response after HRP stimulation, expressed by more than 10 times-fold increase. Natural Api g 5 induced more than two-fold increase. Celery tuber and seed extracts induced similar responses (approx. three-fold increase). Low activation was observed after incubation with rBet v 1 and rApi g 1, respectively (Fig. 3A) and was in accordance with low anti Bet v 1 and anti Api g 1-specific IgE antibodies. RBL assay performed with serum nr 2 confirmed previously observed overall lower levels of specific IgE and resulted in lower values of basophil activation as compared to results obtained by serum nr 1. HRP induced the strongest response of basophils approx. a two-fold increase. Tuber and seed extracts induced a 1.4- and 0.2-fold increase, respectively. Incubation with rApi g 1 did not activate basophils (Fig. 3B).

**Fig. 3:**
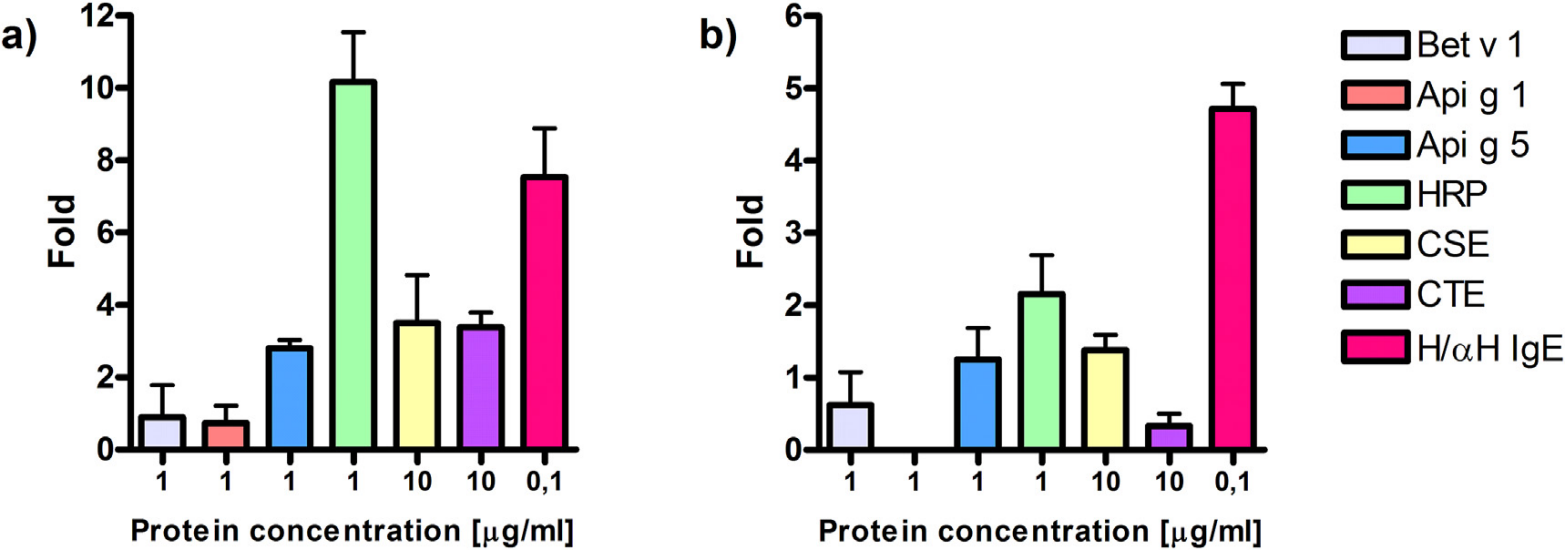
**RBL Assay**. Luciferase assay was performed with RS-ATL8 RBL cells and sera from patient 1 (panel a) and patient 2 (panel b). Human and anti-human IgE (H/αH IgE) were used as a positive control. Luciferase expression levels represented by light units were calculated as a fold increase. Fold = (sample value - spontaneous release value)/spontaneous release value. Results obtained from sera from two patients are presented on a and b, respectively.

For more than 2 decades the role of carbohydrates in an allergic disease has been controversial. Lack of clinical relevance of IgE-binding plant glycans was demonstrated, for example, using human lactoferrin expressed in rice as a model.^13^ All patients (5 in total) with positive basophil activation test did not develop symptoms after SPT and double blind placebo controlled food challenge (DBPCFC). Taking into account small groups of patients, we think that it is still worth considering CCD as a possible allergic factor. Here we report 2 clinical cases with possible role of plant derived CCDs in triggering allergic reaction.

Different protein patterns suggest differences in the content and quantity of potential allergens. This assumption was confirmed by different IgE recognition patterns in immunoblots for both patients’ sera. Additionally, sera blocked with either tuber or seed extract reacted differently in inhibition ELISA assays. Incubation with celery tuber and seed extracts, gives similar activation level of basophils in case of first serum. The second serum caused inequality in basophils activation, and may also indicate a distinction in allergen contents between tuber and seeds. This phenomenon was already shown, ie, for kiwifruit extracts from pulp and seeds, respectively, differing in allergen quantity and quality.^14^ These data indicate that further proteomic studies are needed to assess the allergen profile of different plant organs and tissues.

In our study results obtained from immunoblots and inhibition assays provide evidence that both sera contain specific IgE antibodies directed against cross-reactive carbohydrate domains. In the case of patient number 1, complete inhibition on ELISA and immunoblot suggest, that all IgE antibodies were specific for CCDs while serum from patient number 2 had additional antibodies specific for the major celery allergen, Api g 1.

Interestingly, the protein responsible for cross reactivity between celery and mugwort is still missing. Limited blocking by antibodies specific for a celery extract by nArt v 1 protein may be a partial explanation of this phenomenon. It is worth noticing that the major mugwort allergen, Art v 1, is characterized by O-glycosylation, which may have an effect on the allergenicity.^15^

To sum up, clinical relevance of sIgE directed against CCDs in celery allergy is controversial. However, individual cases highlight it is worth to include them for *in vitro* diagnosis. Differences between the allergen content in different parts of celery should be also considered as a part of the standardized component resolved diagnosis.

## Abbreviations

CCDs, cross-reactive carbohydrate determinants; CRD, component resolved diagnosis; HMW, high molecular weight; RBL, rat basophil leukemia; HRP, horseradish peroxidase; CTE, celery tuber extract; CSE, celery seed extract; SIC, self inhibition control; OD, optical density; BC, buffer control;, NHS, non allergic human serum.

## Funding

This work is partially funded by the 10.13039/501100002428Austrian Science Fund (FWF): DK W 1248-B13 for P.H. and P.D. and FWF: SFB F4603 for K.H.-S.

## Authors contribution

The project outline was drafted by PD, PH and KHS. The experiments were performed by PH, PD, MB, CH, FB, GP, SV, CM-F and KHS. Data analysis and interpretation was done by PH, PD, MB, FB, GP, and KHS. All the authors (PD, PH, MB, CM-F, SV, CH, FB, GP, and KHS) provided input to drafting the article and revising it critically for important intellectual content and gave final approval of the version to be published.

## Ethics statement

The study was approved by the ethics committees of Lower Austria (GS4-EK-4/242-2013) and from the French Ministère de la Recherche (DC-2010-1095). Written informed consents were received from all individuals.

## Consent for publication

All authors consented for publication in this study. Authors confirm that the manuscript is original, has not been published before, is not currently being considered for publication elsewhere, and has not been posted to a preprint server.

## Availability of data and materials

Contact the corresponding author for questions regarding data and materials.

## Declaration of competing interest

The authors declare that they have no competing interests.

## Acceptance of editorial policy

Authors confirm acceptance of editorial policy.
